# Health-Related Quality of Life in People Living with HIV in Southwest Iran in 2018: A Cross-Sectional Study

**DOI:** 10.1155/2021/9935175

**Published:** 2021-08-16

**Authors:** Hassan Joulaei, Seyed Ghaleb Mousavi, Zohre Foroozanfar, Tayebeh Rakhshani

**Affiliations:** ^1^Health Policy Research Center, Institute of Health, Shiraz University of Medical Sciences, Shiraz, Iran; ^2^MA in Community-Oriented Epidemiological Education, School of Health, Shiraz University of Medical Sciences, Shiraz, Iran; ^3^Research Center for Health Sciences, Institute of Health, Shiraz University of Medical Sciences, Shiraz, Iran

## Abstract

Health-related quality of life (HRQoL) is one of the most important indicators in assessing the health and well-being of HIV-positive patients. The present study investigated the HRQoL of HIV patients referred to Abadan's Voluntary Counseling and Testing (VCT) center in 2019. In this cross-sectional study, a total of 134 HIV^+^ patients referred to Abadan's VCT center were selected through convenience sampling. Demographic information was collected through a researcher-made checklist; the patients' status and health information were collected through electronic medical records of HIV^+^ patients and their records at the VCT center. The HRQoL index was assessed using the World Health Organization (WHOQOL-BREF) questionnaire. Data analysis was carried out using simple and multiple linear regression as well as a *t*-test in SPSS software. A *P* value < 0.05 was considered as the significance level in all tests. The mean of the HRQoL in all the participating patients was 56.42 ± 22.66. The highest and lowest mean scores of HRQoL domains were related to social relationships (57.53 ± 24.73) and environmental health (53.68 ± 19.07). There was a positive significant relationship between the marital status, residency, years of education, duration of infection, transmission route, and antiretroviral (ARV) therapy with the score of the HRQoL. The results showed a moderate score for the mean HRQoL and its domains. The present study revealed the necessity of improving HIV^+^ patients' living conditions, employment status, health education, and mental health care.

## 1. Introduction

Quality of life (QoL) has a wide range of contexts and is a complex concept that is affected by physical health, mental status, personal beliefs, social relationships, and environmental factors [1]. The World Health Organization (1974) defined QoL as an individual's perception of his/her position in life in the context of the culture and value systems in which he/she lives and their goals, expectations, standards, and basic issues [2]. According to another definition, health-related quality of life is assessed based on the impact of deficiencies, person's performance and feeling about him/herself, and social opportunities [3].

Studies have shown how social conditions, individual and cultural values, and clinical factors affect QoL [4, 5]. Assessment of health-related quality of life (HRQoL) is an important indicator in people with chronic diseases [6]. HRQoL has been the focus of many clinical researches in recent years. It also plays an important role in evaluating the effect of chronic disease and its treatment on the patients [7]. QOL is also one of the key factors to evaluate the health status of PLWH, and its improvement is one of the important goals of treatment. Assessing the QOL can provide an accurate assessment of how patient life is affected by diseases and treatments [8].

The presence of chronic diseases such as AIDS has a negative impact on HRQoL [9]. AIDS is a disease that affects not only the physical health but also the mental and social conditions of the patients, due to the negative attitude of the society, discrimination, and stigma, especially in developing countries [10]. Evidence suggests that in addition to the underlying infection, social circumstances, relationship issues, comorbidities, and stigma may have an impact on the HRQoL of people with HIV [11]. Stigma is a complex social process of prejudice and negative attitudes towards PLWHA [12]. Despite numerous efforts to reduce the negative effects of HIV-related stigma on the QOL among PLWHA, patients are still facing stigma as a common phenomenon in a variety of contexts, including family, society, workplace, and healthcare settings [13].

Usually, to assess the QoL, a 26-item WHOQoL-BREF is being applied which is translated in different languages including Persian [14]. This questionnaire evaluates three domains of individual health comprising physical, social, mental, and also environmental through 7, 3, 6, and 8 questions, respectively. In addition, it has two more questions that evaluate the individual perception about her/his health status and quality of life in general [15].

The results of a study implemented in Tehran using WHOQoL-BREF showed that patients with HIV are faced with many problems when trying to get medical treatment for the disease and other conditions because of stigma, lack of awareness, incomplete information on how the disease is transmitted, or intense fear of personnel and health professionals [16]. Experiencing HIV-related stigma and discrimination among PLHIV is associated with a number of adverse health outcomes including feelings of anxiety, fear, frustration, depression, stress, shame, rejection, and self-isolation as well as limited access to treatment and healthcare services [17]. Improving the quality of life is central to the care and support of PLHIV [18]. HRQoL assessment, in fact, shows how the disease and comprehensive treatment have affected the quality of life of HIV^+^ patients [6]. The results of a meta-analysis of 28 studies showed that comprehensive treatment strategies and provision of diverse and continuous care models including treatment, physical, mental, and psychological rehabilitation services and home-based services can improve the outcomes of the disease and have a positive effect on HRQoL of these patients [19].

Poor quality of services or lack of access to services in PLHIV facing treatment problems can produce challenges related to adherence, potentially increase the risk of disease transmission, and reduce HRQoL [20]. The results of previous studies have shown that providing comprehensive treatment measures, including psychological and physical rehabilitation as well as home-based care, yields a good outcome for these patients and also plays an effective role in their HRQoL [6].

Regarding the diverse sociocultural context of Iran and the effect of this diversity on HRQoL on the one hand and our limited knowledge about the QoL of PLHIV in Abadan with different sociocultural contexts on the other hand, this study is conducted for the first time to assess this important health indicator. Through this study, we sought to determine not only the physical and therapeutic needs of PLHIV but also their psychological, social, environmental, and spiritual needs. Determining these needs can help the physicians choose the best and effective treatment [21]. Hence, the aim of this study was at evaluating the quality of life and its determinants amongst PLHIV referred to the VCT center in Abadan, southwest of Iran. It also examined the association between HRQoL and their health status.

## 2. Materials and Methods

### 2.1. Study Context and Sampling Method

This descriptive analytical cross-sectional study was performed on HIV^+^ patients referred to the VCT center of Abadan in 2019. Abadan is an oil city located in the southwest of Iran with around 298090 population. There is one VCT center in Abadan with 281 registered patients who are receiving regular health care. The expected sample size was calculated to be 137 individuals using Cochran's method. After data collection, three incomplete questionnaires were deleted and these people were excluded from the study and the final sample size was 134 cases. The sample size was calculated using equation (1) as follows:

(1) $$\begin{array}{c} N=\frac{\left(t^{2}pqn\right)}{\left(n-1\right)d^{2}+t^{2}pq},\\ N=\frac{\left(1.96^{2}\times 0.5\times 0.5\times 281\right)}{\left[\left(281-1\right)0.06^{2}+1.962\times 0.5\times 0.5\right]}=137. \end{array}$$

The participants were selected using the convenience sampling method in the sense that the research tools were performed on all patients who referred to the VCT center of Abadan until the samples reached 137. Three participants have not answered to more than half of the questions. So, the investigator called them two times to invite them to complete the questionnaire or ask the questions by telephone, but they denied. Therefore, the three incomplete questionnaires, which may affect the results of the study, were omitted.

### 2.2. Inclusion and Exclusion Criteria

The inclusion criteria were patients aged over 18 years, having a positive Western blot test from the beginning of 2004 to the end of 2016, having two consecutive positive ELISA tests from the beginning of 2017 (fourth generation), and having a medical case and code in the VCT center. Exclusion criteria also included unwillingness to participate in the study or severe cognitive impairments such as mental retardation confirmed by a psychiatrist.

## 3. Data Collection Tools and Method

To assess the relationship between HRQoL and its contributing factor in PLHIV, we used a two part tool. The first part included demographic and health status information. Demographic information included age, sex, place of residence, marital status, level of education, and occupation. Health status information, included disease duration, route of transmission, initiation of ARV therapy, CD4 count at the time of the study, history of injecting drug use, current drug use, and clinical stage of the disease. To gather these information, we used electronic medical records of the PLHIV in Abadan's VCT center.

The second part included HRQoL information. To gather this information, a 26-item WHOQOL-BREF was applied. It encompasses 4 areas including physical, psychological, social, and environmental health. The developers of this scale reported acceptable reliability by the internal consistency method and reported Cronbach's alpha coefficient of 73%–98% [22]. This questionnaire was also translated into Persian by Iranian researchers and its validity and reliability were confirmed by Nedjat et al. [23] for the first time [23]. In the present study, the reliability of the whole questionnaire and physical health, psychological health, social relationships, and environmental health of the QoL was 0.94, and 0.83, 0.79, 0.75, and 0.84, respectively. The total score and scores for physical health, psychological health, social relationships, and environmental health were assigned based on a Likert scale. Initially, a raw score is obtained for each domain, which must be converted to a standard score between 0 and 100 or 0 and 20 through a special score conversion formula or table. A higher score indicates a higher HRQoL [15]. However, based on the instrument's guideline, questions number 3, 4, and 26 asked about negative feeling so they are not scored in a positive direction, i.e., higher scores do not denote a higher quality of life.

To collect the required information in this part, we provided a private room in the VCT center and let the PLHIV to fill the self-administered 26-item WHOQOL-BREF. The information was completed with the cooperation of the skilled staff of the VCT center due to more trust of the patients in them and their previous communication and contacts. In case of illiteracy of the participants, it is completed by the VCT staff through an interview with them.

## 4. Statistical Analysis

Data analysis was performed using descriptive statistics. Qualitative variables were described as the number and percentage, and quantitative variables were reported in terms of mean and standard deviation. Statistical *t*-test was used to determine the difference between the score of each dimensions of the questionnaire in the subgroups of treatment (yes or no), the duration of the disease (less than 5 years or higher than 5 years), and the route of transmission (sexual or injection). In order to determine the contribution of the demographic variables and health status as a predictor of HRQoL (criterion variable) in HIV-positive patients, linear regression analysis was used. First, simple linear regression analysis was used to determine the factors associated with QoL of HIV-positive patients, and then, the variables that were *P* ≤ 0.2 in simple analysis were entered into the multiple linear regression model.

It should be noted that before performing the above process, the assumptions associated with a linear regression model were tested by Spearman correlation analysis to check the correlation and the independence of errors using the Durbin-Watson test. To examine the correlation, which indicates that an independent variable is a linear function of other independent variables, we calculated the tolerance parameter and the variance inflation factor (VIF). Data were analyzed by STATA software version 12 and SPSS software version 24. In all tests, a *P* value < 0.05 was considered as the level of statistical significance.

## 5. Ethical Consideration

All steps of the research process were performed confidentially without mentioning the patient's name and using a patient-specific code. The patients were also informed about the objectives of the research, confidentiality of their information, and voluntary participation and after completing the informed consent that they received to respond to the questionnaire. This study was approved by the ethics committee of the research deputy for Shiraz University of Medical Sciences with the code of IR.SUMS.REC.1397.974.

## 6. Results

In the current study, 134 patients with HIV were enrolled; of them, 99 (67.2%) were male. The mean age of the participants was 37.86 ± 9.02 years. Demographic characteristics and disease status of the patients were stratified by sex, as listed in Table 1.

**Figure 1:**
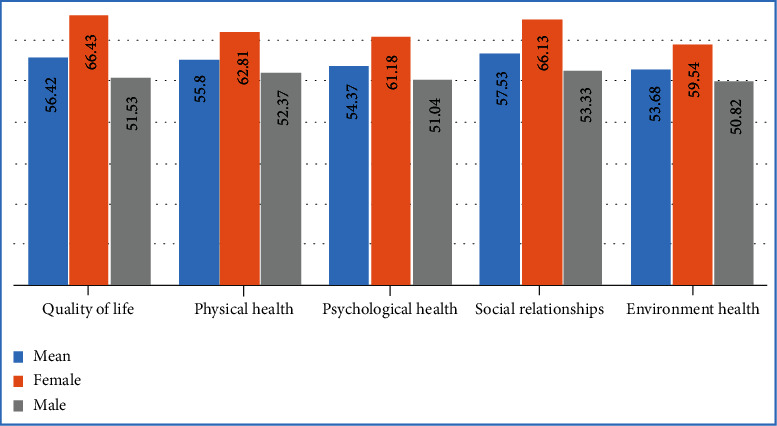
Mean of total scores of quality of life and its four domains based on HIV^+^ patients' gender.

Results showed that the mean of HRQoL in all participating patients was 56.42 ± 22.66. Regarding the HRQoL domains, the mean score of all domains is as follows: social relationships (57.53 ± 24.73), environmental health (53.68 ± 19.07), physical health (55.80 ± 20.20), and psychological health (54.37 ± 19.30); the highest and lowest mean scores were related to social relationships and environmental health, respectively.  Figure 1 demonstrates a comparison of the overall mean scores of HRQoL and its domains among women and men. Women had higher scores in the HRQoL in the four domains of physical health (62.81 ± 16.87), psychological health (61.18 ± 13.13), environmental health (59.54 ± 12.96), and social relationships (66.13 ± 17.54) than men.

The results of simple linear regression analyses showed that the factors, as shown in Table 2, including sex, marital status, employment status, duration of infection, transmission route, antiretroviral therapy, and history of drug injection, were associated with the QoL score. Women, married and widowed/divorce patients, housewives, people with a duration of infection of more than 5 years, sexual and unknown transmission route, and treatment with antiretroviral had statistically a positive relationship with higher scores of QoL, but the history of drug injection was associated with lower scores of HRQoL.

Results showed higher scores of social relationships and environmental health domains (*P* < 0.05) among antiretroviral treatment recipients. However, there was no significant difference between the two groups in terms of physical and psychological health (*P* > 0.05). Moreover, investigation of the difference in the disease duration showed higher scores of the four domains in HIV-infected people up to 5 years and this difference was significant in all domains except environmental health (*P* < 0.05). The HRQoL score was significantly lower among patients getting HIV through injecting drugs than those having sex and its four domains in people with a history of injecting drugs than those without such a history (*P* < 0.05).

According to multiple linear regression, there was a statistically significant relationship between the marital status, residency, years of education, duration of infection, transmission route, antiretroviral therapy, and HRQoL score. Married patients compared to single, people with university education compared to illiterate, rural patients compared to urban patients, people with a duration of infection of more than 5 years compared to less than 5 years, and unknown transmission route compared to injection and treatment with antiretroviral compared to no treatment had a statistically positive relationship with higher scores of HRQoL (Table 3).

## 7. Discussion

The mean HRQoL score of all participants was moderate (56.42) which is comparable with the general population of less or partially developed areas of Abadan [24]. The highest and the lowest mean scores were obtained in the social relationships and environmental health domains, respectively. The present study showed that women had higher scores in HRQoL in the four domains of physical health, psychological health, environmental health, and social relationships than men; this is inconsistent with previous studies by Cederfjall et al. in Sweden [25], Sabermanian et al. in India [26], and Nojoomi et al. in Iran [27]. However, the questionnaire used in Sederfjal et al.'s and Sabermanian et al.'s studies was different from the one used in the present study. Another study carried out in Tehran revealed that women had higher scores in physical and social domains. The possible interpretation of this finding could be the effect of addiction on the QoL. As our results showed being in line with Seresht et al.'s study, the rate of addiction and its history in female are significantly lower in comparison with those in men. [28].

As mentioned in the study in Tehran, HIV-infected drug users had lower HRQoL than non-HIV addicts [29]. A study in Massachusetts also found ample evidence suggesting that addiction alone had increasing negative effects on HRQoL of healthy individuals and that these problems were more prevalent in HIV-infected patients [30]. Noticeably, in the present study, 100% of the current drug users were men. Also, 95% of men reported the history of injecting drug use, which may have a great impact on their HRQoL, thereby reducing their mean HRQoL score. In line with the present study, other domestic or foreign studies also reported that males outnumber females among drug users. Married people had a higher HRQoL than single and widowed people [26–28, 31]. These people also reported higher life pleasure and sexual satisfaction. However, Razavi et al. reported no relationship between the marital status and HRQoL [32].

According to our study, the ARV-treated group had significantly higher HRQoL scores in social relationships and environmental health domains than the untreated group. Satisfaction with healthcare services was also higher in these people. Likewise, studies implemented in Uganda, Morocco, Zimbabwe, and Georgia showed similar findings [33–36].

There was a relationship between the disease transmission route and QoL, and people who were infected through injection had a lower HRQoL than people infected through sex; this was not consistent with similar studies conducted in Iran or Brazil [28, 32, 37]. The reason for this difference could be a higher number of women infected through sex, which has a significant impact on this index, considering higher HRQoL in women.

There is a controversy in the relationship between CD4 counts with HRQoL; on the one hand, our study, in line with several other studies, did not find a significant relationship [6, 38]. On the other hand, some evidence from Iran and Uganda showed that this relationship was significant [27, 33]. Differences in the sociocultural context in terms of stigmatization and healthcare settings in terms of quality of care would be an interpretation for this controversy.

Furthermore, in line with other evidence in Iran, our results revealed that employed people had a higher HRQoL score than the unemployed ones and also the employed people [6, 28]. Likewise, a direct and significant relationship has been shown between the level of education and HRQoL [6, 27]. It seems that good financing of patients with HIV, due to their employment status, would improve their HRQoL. Also, the level of education might be a worthy determinant for better coping with their new life after receiving HIV. A study performed in Italy found that economic problems and concerns about treatment costs are major concerns of HIV-positive patients and the higher the level of concern, the lower the HRQoL and the more severe the symptoms will be [39].

Given that, in Iran, all healthcare delivered in the VCTs is free, 62.7% of the participants had good and very good satisfaction about healthcare services, which can be attributed to the appropriate medical support of patients referred to this center. Another study from Iran also reported that healthcare satisfaction increased significantly in patients and this issue played an important role in improving HRQoL scores of patients [40]. Bangston et al. also stated in their study that improving access to the necessary care will increase the HRQoL among HIV-positive people [41].

Another controversy is found in the relationship between the clinical stage of the disease and patients' HRQoL. While some evidence from Iran and West Africa revealed no significant relationship between the clinical stage and HRQoL [6, 27, 42], other in-reach evidence from Iran, Croatia, and Brazil, in line with our findings, approved a significant relationship [28, 32, 43, 44]. Noticeably, specialized tests such as viral load, which is a good decision criterion for determining the clinical stage of the disease, were not performed for most patients in the present study.

Implemented studies in Iran [16, 45] revealed that stigma would be a main barrier for utilization of healthcare services by PLHIV and it might affect their quality of life. Hence, reducing stigma could be an effective strategy to improve their HRQOL. To approach, this strategy effectively, legislators, and policy-makers should be involved.

## 8. Strengths and Limitation

Although similar studies are published in different areas of Iran, given cultural diversity and different levels of quality of care delivered to HIV^+^ patients in Iran, implementing this study in Abadan for the first time is one of its strengths. However, it had some limitations such as hard direct access to these patients, low level of education, and unwillingness to participate for some of them. The researchers did their best to solve these barriers through involvement of a skilled staff of the VCT center in interviews and pay the participants some money, to compensate their time and commuting cost and to encourage them to participate.

## 9. Conclusion

The results of the current study revealed a moderate HRQoL score for physical, social, and psychological domain and the lowest scores for the environmental health domain. To establish effective strategies, an additional study considering the findings of the current study and other similar studies and with large samples is recommended. Also, a further study is recommended to define the relationship between social stigma and the patients' HRQoL. Moreover, based on our findings, improving psychological counseling services and patients' social relationship by including their families in counselling services would be helpful to enhance their QoL.

## Figures and Tables

**Table 1:** Characteristics of HIV^+^ patients referred to Abadan's VCT center.

Characteristics	Total (*n* = 134)	Men (*n* = 90)	Women (*n* = 44)
*Age*	37.86 ± 9.02	39.52 ± 9.03	34.45 ± 8.06
*Age (years)*			
≤30	30 (22.4)	13 (14.4)	17 (38.6)
31-40	57(42.5)	39 (43.3)	18 (40.9)
41-50	37 (27.6)	32 (35.6)	5 (11.4)
51≤	10 (7.5)	6 (6.7)	4 (9.1)
*Marital status*			
Never married	34 (25.4)	34 (37.8)	—
Married	66 (49.2)	40 (44.8)	26 (59.1)
Widowed/divorce	34 (25.4)	16 (17.8)	18 (40.9)
*Residency*			
Urban	114 (85.1)	82 (91.1)	32 (72.7)
Rural	20 (14.9)	8 (8.9)	12 (27.3)
*Years of education*			
0	8 (6.0)	6 (6.7)	2 (4.5)
1–5	22 (16.4)	12 (13.3)	10 (22.7)
6–8	56 (41.8)	46 (51.1)	10 (22.7)
9–12	40 (29.9)	20 (22.2)	20 (45.5)
>12	8 (6.0)	6 (6.7)	2 (4.5)
*Employment status*			
Unemployed	32 (23.9)	30 (30.3)	2 (4.5)
Employed	62 (46.2)	60 (66.7)	2 (4.5)
Housewife	40 (29.9)	—	40 (90.9)
*Duration of infection*			
Less than 5 years	88 (65.7)	62 (68.9)	26 (59.1)
More than 5 years	46 (34.3)	28 (31.1)	18 (40.9)
*Transmission route*			
Injection	70 (52.2)	70 (77.8)	—
Sexual	44 (32.8)	10 (11.1)	34 (77.3)
Unknown	20 (14.9)	10 (11.1)	10 (22.7)
*Antiretroviral therapy*	124 (92.5)	80 (88.9)	44 (100.0)
*CD4 cell count*			
Less than 350	54 (40.3)	42 (46.7)	12 (27.3)
More than 350	80 (59.7)	48 (53.3)	32 (72.7)
*Drug use*	22 (16.4)	22 (24.4)	—
*History of drug injection*	80 (59.7)	76 (84.4)	4 (9.1)
*Clinical stage*			
Asymptomatic	98 (73.1)	64 (71.1)	34 (77.3)
Symptomatic	24 (17.9)	18 (20.0)	6 (13.6)
AIDS	12 (9.0)	8 (8.9)	4 (9.1)

Qualitative variables are reported as a number (percentage) and quantitative variables as mean ± standard deviations.

**Table 2:** Factors associated with the quality of life in HIV^+^ patients referred to Abadan's VCT center; simple analysis.

	*B*	SE	*P* value
*Age*	0.07	0.22	0.749
*Sex*			
Male	1		
Female	14.89	3.97	0.001^∗^
*Marital status*			
Never married	1		
Married	15.04	4.63	0.002^∗^
Widowed/divorce	10.97	5.33	0.041^∗^
*Residency*			
Urban	1		
Rural	10.02	5.44	0.068
*Years of education*			
0	1		
1–5	5.47	9.25	0.555
6–8	7.16	8.47	0.400
9–12	16.22	8.68	0.064
>12	15.75	11.21	0.162
*Employment status*			
Unemployed	1		
Employed	−5.02	4.72	0.289
Housewife	11.51	5.14	0.027^∗^
*Duration of infection*			
Less than 5 years	1		
More than 5 years	18.25	3.82	0.001^∗^
*Transmission route*			
Injection	1		
Sexual	14.77	4.18	0.001^∗^
Unknown	11.31	5.51	0.042^∗^
*Antiretroviral therapy*			
No	1		
Yes	25.96	7.13	0.001^∗^
*CD4 cell count*			
Less than 350	1		
More than 350	3.22	3.99	0.421
*Drug use*			
No	1		
Yes	−2.41	5.30	0.650
*History of drug injection*			
No	1		
Yes	−13.89	3.82	0.001^∗^
*Clinical stage*			
Asymptomatic	1		
Symptomatic	-6.30	5.15	0.223
AIDS	-8.38	6.91	0.227

^∗^Significant at 0.05 level.

**Table 3:** Factors associated with the quality of life in HIV^+^ patients referred to Abadan's VCT center; multiple analysis.

	*B*	SE	*P* value
*Age*	0.164	0.20	0.421
*Marital status*			
Never married	1		
Married	13.76	4.56	0.003^∗^
Widowed/divorce	6.47	5.12	0.209
*Residency*			
Urban	1		
Rural	11.97	5.39	0.028^∗^
*Years of education*			
0	1		
1–5	8.15	8.21	0.322
6–8	9.24	7.43	0.216
9–12	11.80	7.30	0.109
>12	22.05	9.75	0.026^∗^
*Duration of infection*			
Less than 5 years	1		
More than 5 years	22.02	3.56	0.001^∗^
*Transmission route*			
Injection	1		
Sexual	6.46	4.62	0.164
Unknown	10.71	5.09	0.038^∗^
*Antiretroviral therapy*			
No	1		
Yes	21.15	6.42	0.001^∗^

^∗^Significant at 0.05 level.

## Data Availability

All data and materials generated in this study are included in this article, but if necessary, other data will be made available from the responsible author upon request.

## Abbreviations

QoL: Quality of life
HRQoL: Health-related quality of life
VCT: Voluntary counseling and testing
ARV: Antiretroviral
WHOQOL-BREF: World Health Organization
VIF: Variance inflation factor.

## Additional Points

*Publisher's Note.* Springer Nature remains neutral with regard to jurisdictional claims in published maps and institutional affiliations.
